# Identification of Dipeptidyl Peptidase-4 and α-Amylase Inhibitors from *Melicope glabra* (Blume) T. G. Hartley (Rutaceae) Using Liquid Chromatography Tandem Mass Spectrometry, In Vitro and In Silico Methods

**DOI:** 10.3390/molecules26010001

**Published:** 2020-12-22

**Authors:** Alexandra Quek, Nur Kartinee Kassim, Amin Ismail, Muhammad Alif Mohammad Latif, Khozirah Shaari, Dai Chuan Tan, Pei Cee Lim

**Affiliations:** 1 Department of Chemistry, Faculty of Science, Universiti Putra Malaysia, Serdang, Selangor 43400, Malaysia; quek.alexandra@gmail.com (A.Q.); aliflatif@upm.edu.my (M.A.M.L.); khozirah@upm.edu.my (K.S.); tandaichuan@yahoo.com (D.C.T.); 2 Integrated Chemical BioPhysics Research, Faculty of Science, Universiti Putra Malaysia, Serdang, Selangor 43400, Malaysia; 3 Department of Nutrition and Dietetics, Faculty of Medicine and Health Sciences, Universiti Putra Malaysia, Serdang, Selangor 43400, Malaysia; aminis@upm.edu.my; 4 Natural Medicines & Products Research Laboratory, Institute of Bioscience, Universiti Putra Malaysia, Serdang, Selangor 43400, Malaysia; 5 Faculty of Pharmacy, Mahsa University, Bandar Saujana Putra, Jenjarom, Selangor 42610, Malaysia; pclim@mahsa.edu.my

**Keywords:** *Melicope glabra*, antidiabetic, diabetes, DPP-4, α-Amylase, molecular docking, flavonoids, phenolics

## Abstract

The present study investigated the antidiabetic properties of the extracts and fractions from leaves and stem bark of *M. glabra* based on dipeptidyl peptidase-4 (DPP-4) and α-Amylase inhibitory activity assays. The chloroform extract of the leaves was found to be most active towards inhibition of DPP-4 and α-Amylase with IC_50_ of 169.40 μg/mL and 303.64 μg/mL, respectively. Bioassay-guided fractionation of the leaves’ chloroform extract revealed fraction 4 (CF4) as the most active fraction (DPP-4 IC_50_: 128.35 μg/mL; α-Amylase IC_50_: 170.19 μg/mL). LC-MS/MS investigation of CF4 led to the identification of trans-decursidinol (**1**), swermirin (**2**), methyl 3,4,5-trimethoxycinnamate (**3**), renifolin (**4**), 4′,5,6,7-tetramethoxy-flavone (**5**), isorhamnetin (**6**), quercetagetin-3,4′-dimethyl ether (**7**), 5,3′,4′-trihydroxy-6,7-dimethoxy-flavone (**8**), and 2-methoxy-5-acetoxy-fruranogermacr-1(10)-en-6-one (**9**) as the major components. The computational study suggested that (**8**) and (**7**) were the most potent DPP-4 and α-Amylase inhibitors based on their lower binding affinities and extensive interactions with critical amino acid residues of the respective enzymes. The binding affinity of (**8**) with DPP-4 (−8.1 kcal/mol) was comparable to that of sitagliptin (−8.6 kcal/mol) while the binding affinity of (**7**) with α-Amylase (−8.6 kcal/mol) was better than acarbose (−6.9 kcal/mol). These findings highlight the phytochemical profile and potential antidiabetic compounds from *M. glabra* that may work as an alternative treatment for diabetes.

## 1. Introduction

In recent times, owing to their therapeutic and marketable properties, the search for natural bioactive compounds from plants has been the target for the food and pharmaceutical industries. Dietary plants were reported to act on diverse targets of metabolic maladies through different mechanisms and modes intended for distinctive therapeutic effects in the disease and/or its complications. The literature has reported many plants with therapeutic properties and Rutaceae plants are one of the medicinal plants with widely described bioactivities, including antidiabetic [1], antibacterial [2], anticancer [3], and anti-inflammatory activities [4]. *Melicope glabra* (Blume) T. G. Hartley, a plant belonging to Rutaceae, is among the significant sources of flavonoids and coumarins [5,6]. It is commonly called “pepauh daun besar” or “tenggek burung” in local Malaysian folklore, and the leaves of the plant are consumed for general well-being. Additionally, the plant was also known for its effectiveness against cough and fever. Various plant phenolics have been known as effective antidiabetic compounds. For instance, plant phenolics such as quinic acid, rutin, kaempferol, emodin, suberosin, and feramidin were reported as natural DPP-4 and α-Amylase inhibitors [7]. Naturally occurring flavonoids such as anthocyanidins, flavanones, isoflavones, and flavonols have been suggested as useful supplements to manage and prevent diabetes as well as its long-term complications by improving the lipid profile, glucose metabolism, and hormone regulations in the human body [8].

Diabetes mellitus is a cluster of metabolic disorders defined and characterized by high blood glucose levels induced by insulin secretion deficiencies or insulin resistance, or a combination of both [9]. If it is left untreated, diabetes mellitus substantially escalate the risk of renal failure, cardiovascular diseases, ischemic heart disease, and other severe health complications [10].

Currently, various oral clinical antidiabetic drugs have been applied to control hyperglycemia. Nonetheless, clinical inhibitors of DPP-4 like sitagliptin, linagliptin, and saxagliptin, and α-Amylase inhibitors like acarbose, voglibose, and miglitol, are associated with unfavorable side effects such as gastrointestinal discomfort, pancreatitis, diarrhea, and congestive heart failure [11,12]. These reported adverse effects of clinical drugs also led to poor medication adherence among many diabetic patients, who preferred natural product consumption [13]. Therefore, novel inhibitors of these enzymes from natural sources with lesser side effects are of interest.

The objectives of this study were thus to find potential antidiabetic compounds from *M. glabra* via dipeptidyl peptidase-4 (DPP-4) and α-Amylase inhibitory assay-guided fractionation approaches. Phytochemical profiling and in silico molecular docking were performed on the most active fraction to tentatively identify the antidiabetic compounds. To the best of our knowledge, this is the first study that investigated antidiabetic activities of different plant parts of *M. glabra* and its phytochemical profile.

## 2. Results and Discussion

### 2.1. Extraction Yield

Solvents of different polarities, including hexane, chloroform, and methanol, were used to maximize phytocomplex extraction from *M. glabra* leaves and stem bark samples. The extraction yield of phytocomplex in different extracts of *M. glabra* is tabulated in Table 1. Significantly, methanol extracts from both samples gave the highest (*p* < 0.05) yield, followed by chloroform and hexane. However, the recovery yield from the leaves of *M. glabra* was higher (*p* < 0.05) than the stem bark. The recovery yields of leaves’ crudes were 2.03 ± 0.31%, 2.79 ± 0.19%, and 7.30 ± 1.10% for hexane leaves, chloroform leaves, and methanol leaves, respectively. Meanwhile, the stem bark crudes gave respective recovery yields of 0.3 ± 0.16%, 0.7 ± 0.03%, and 2.13 ± 0.25% for hexane stem bark, chloroform stem bark, and methanol stem bark. Similar findings can be observed for the other Rutaceae plants [14,15], where the extraction of leaves resulted in a higher amount than that of stem bark. It was comprehended that the variation of phytocompounds in different plant parts closely depends on their function in the lifecycle and growth phase of each plant [16]. In addition, due to the complex nature of plant metabolites, different phytochemicals are recovered in organic solvents of different polarities. In this study, the higher yield obtained from methanol solvent indicated that the extractable constituents of the *M. glabra* were mainly polar to highly polar compounds.

### 2.2. Dipeptidyl Peptidase-4 Inhibitory Activity

Oral inhibitors of enzyme dipeptidyl peptidase-4 (DPP-4) are currently of interest in the treatment of diabetes. They act as antihyperglycemic agents that block the enzyme DPP-4, a serine protease that most often presents in kidneys, the gastrointestinal tract, and the endothelial layer of blood vessels. This enzyme has the ability to deactivate incretin hormones, glucagon-like peptide-1, and glucose-dependent insulinotropic polypeptide, which are essential for insulin production from pancreatic β-cells and glucagon release inhibition from the α-cells [17,18].

The DPP-4 inhibitory potential of *M. glabra* crude extracts is presented in Table 2. Significantly, both chloroform extracts from the leaves and stem bark (*p* < 0.05) possessed the most inhibition against DPP-4 in which the leaves (IC_50_: 169.40 ± 9.30 μg/mL) showed higher inhibition than the stem bark (IC_50_: 332.31 ± 10.07 μg/mL). A similar finding was reported where the chloroform extract of *Inonotus obliquus* had the highest DPP-4 inhibitory activity compared to the hexane, ethyl acetate, and methanol extracts [19]. Chloroform extract is rich in compounds of intermediate polarity, including flavonoids. Flavanoids have been identified as the bioactive compounds that were responsible for effective DPP-4 inhibitions of many herbs such as Mexican oregano, rosemary, and marjoram [20]. Thus, the higher inhibition activity of chloroform extract of *M. glabra* could be similar due to flavonoid components.

Bioassay-guided fractionation of chloroform leaves’ extract afforded five fractions (CF1-CF5) and their inhibition potency against the DPP-4 was investigated. All fractions possessed inhibition against DPP-4 in a concentration-dependent manner with IC_50_ values ranging from 128.35 to 1711.06 μg/mL (*p* < 0.05) (Table 2). The maximum inhibitory activity was observed in CF4 (IC_50_: 128.35 ± 12.77 μg/mL) followed by CF3 (IC_50_: 313.18 ± 20.92 μg/mL) and CF2 (IC_50_: 619.31 ± 9.21 μg/mL). However, no fractions were comparable to the positive control sitagliptin, which showed inhibition with the IC_50_ value of 0.01 ± 0.01 μg/mL.

### 2.3. α-Amylase Inhibitory Activity

α-Amylase is a digestive enzyme responsible for the digestion of carbohydrates. Salivary α-Amylase breaks down α-(1,4)-glycosidic bonds of starch and oligosaccharides into disaccharides during the process of food bolus formation and swallowing [21]. Suppression of post-prandial hyperglycemia can be achieved by inhibition of α-Amylase, which subsequently slows down the digestion of carbohydrates and reduces glucose absorption into the bloodstream.

The α-Amylase inhibitory activity of the *M. glabra* extracts is presented in Table 2. All the plant extracts showed inhibition against α-Amylase with chloroform leaves’ extract being the most active extract (IC_50_: 303.64 ± 10.10 μg/mL). The other plant extracts showed lower inhibition activity with IC_50_ values ranging from 975.80 to 5447.01 μg/mL. Chloroform extract is a potential inhibitor against α-Amylase. This observation was further supported by a study on *Paederia foetida* where the chloroform extract (medium polar) of the plants showed higher α-Amylase inhibition than the higher polar extracts [22].

Among the chloroform leaves’ fractions, CF4 had shown the highest inhibition activity with an IC_50_ value of 170.19 ± 20.66 μg/mL (*p* < 0.05), which was comparable to the positive control, acarbose (IC_50_: 188.60 ± 14.31 μg/mL) (Table 2). This is followed by CF2 (IC_50_: 253.30 ± 19.21 μg/mL) and CF3 (IC_50_: 531.44 ± 11.38 μg/mL). CF5 and CF1 showed relatively milder inhibition.

### 2.4. Phytochemical Profiling of Active Fraction Using LC-MS/MS

**Figure 1:**
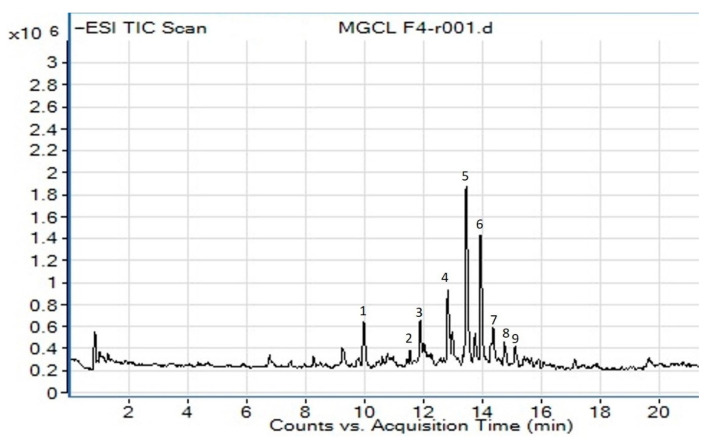
The total ion chromatogram of *M. glabra* chloroform leaves’ crude fraction CF4 obtained from LC-MS/MS under negative ion mode. Compounds are numbered based on retention times (RT). The *x*-axis is the retention time, and the *y*-axis is the intensity of the *m*/*z* peaks.

Phytochemical profiling of the most active fraction, CF4, from chloroform leaves of *M. glabra* was performed using LC-MS/MS analysis and the ChemSpider database. Figure 1 represents the total ion chromatogram of CF4 under negative ionization mode. The constituents were identified through the interpretation of their MS, MS/MS spectra, and comparison with the library data. Notably, nine phenolic compounds were tentatively identified in CF4. The LC-MS/MS data of the compounds identified are listed in Table 3.

**Figure 2:**
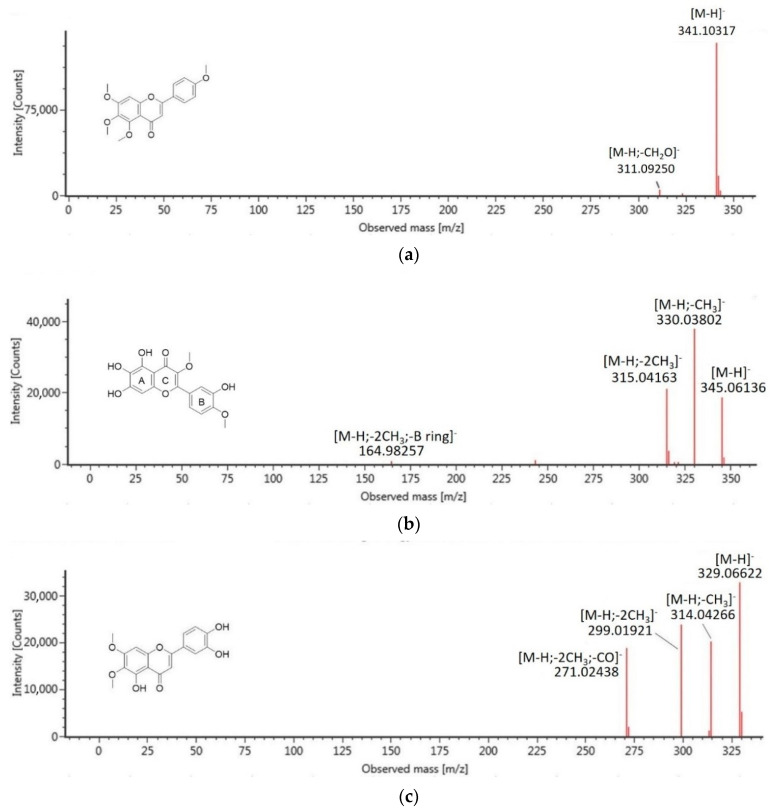

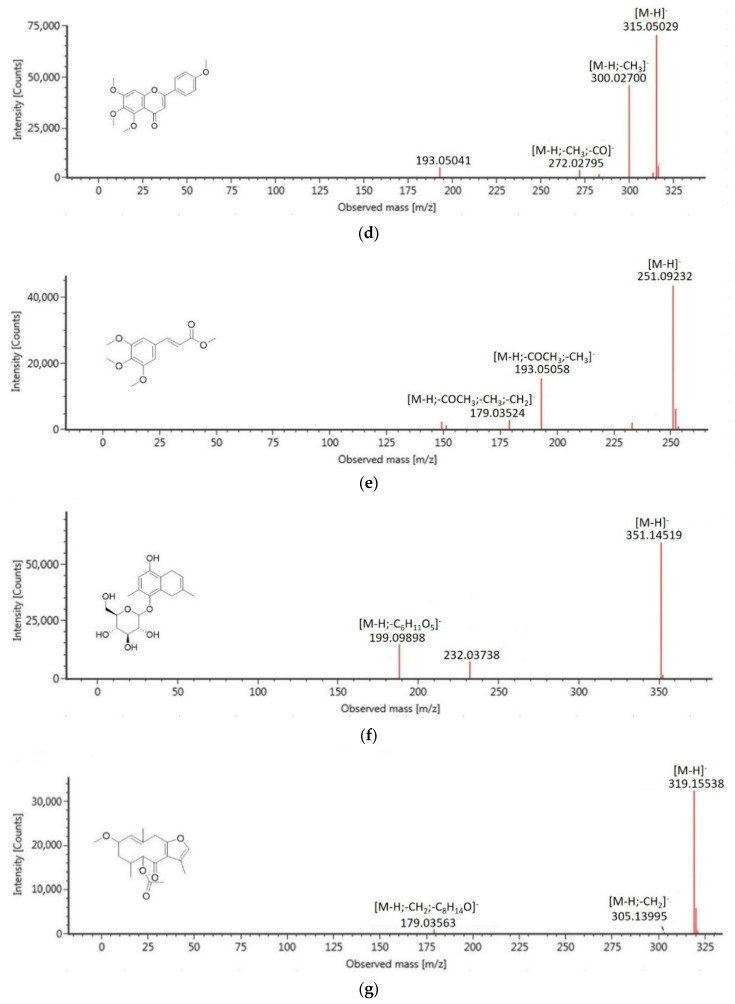

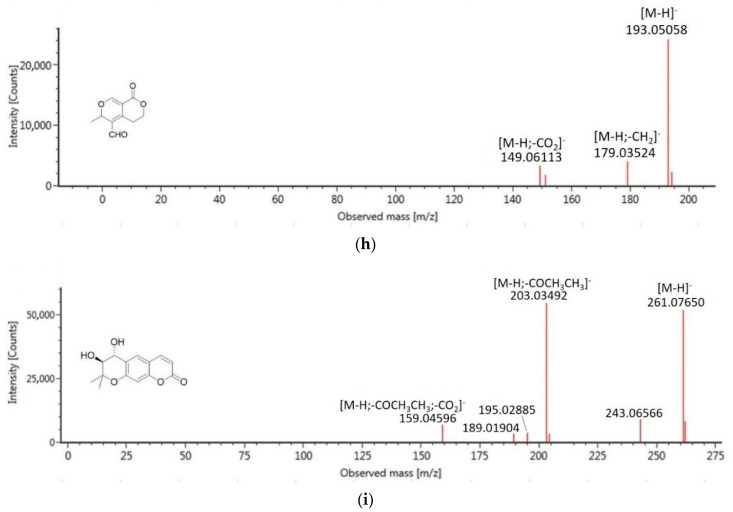
MS/MS spectra fragmentation of tentatively identified compounds from CF4: (**a**) 4′,5,6,7-tetramethoxy-flavone, (**b**) quercetagetin-3,4′-dimethyl ether, (**c**) 5,3′,4′-trihydroxy-6,7-dimethoxy-flavone, (**d**) isorhamnetin, (**e**) methyl 3,4,5-trimethoxycinnamate, (**f**) renifolin, (**g**) 2-methoxy-5-acetoxy-fruranogermacr-1(10)-en-6-one, (**h**) swermirin, (**i**) trans-decursidinol.

Phenolic compounds are a group of natural constituents that are able to provide antidiabetic therapy. These compounds are generally categorized as flavonoids, lignans, stilbenes, coumarins, and others. Of the nine phenolic compounds tentatively identified in CF4, four of them were suggested as flavonoid compounds (**5**–**8**). For many years, dietary flavonoids have been known for their effects on preventing degenerative diseases and are effective for treating diabetes. The antidiabetic efficacy of flavonoids, specifically by inhibition of DPP-4 and α-Amylase, are attributed to their structural characteristics such as the double bond between C2 and C3 of ring C and the catechol structure of ring B [23], which interestingly can be observed in the structure of identified flavonoids in this study. Besides flavonoids, other phenolic compounds that were suggested in CF4 included cinnamic acid derivative, glucoside, lactones, and coumarins. The finding proposed that the significant antidiabetic effects reported in the active extracts and fractions of *M. glabra* were due to the presence of plant phenolic compounds. The identified compounds in CF4 were subjected to in silico molecular docking analysis to investigate their antidiabetic potential further to support the statement. Among the compounds, compounds (**4**), (**5**), and (**6**) are of higher abundance. Compound (**4**) with molecular ion [M−H]^−^ at *m*/*z* 351 and a fragment ion at *m*/*z* 199 [M-H-C_6_H_11_O_5_]^−^, due to the breakage of the glycosidic bond, was typically assigned as a glucoside compound, renifolin. Meanwhile, compounds (**5**) and (**6**) had fragmentation patterns that suggested them as flavonoid compounds. Compound (**5**) with molecular ion [M−H]^−^ at *m*/*z* 341 has been assigned as 4′,5,6,7-tetramethoxy-flavone whereby its major fragment ion at *m*/*z* 311 represented the loss of CH_2_O, which occurred at the methoxy group at the 4′ position of the parent ion [24]. Compound (**6**), which produced molecular ion [M-H]^−^ at *m*/*z* 315, was assigned as isorhamnetin, of which its major fragment ions at *m*/*z* 300 and *m*/*z* 272 were attributed to the subsequent loss of CH_3_ and CO. Figure 2 summarizes the MS/MS spectra fragmentation of the identified compounds analyzed to confirm their suggested structures.

### 2.5. In Silico Inhibitory Analysis of Identified Compounds

**Figure 3:**
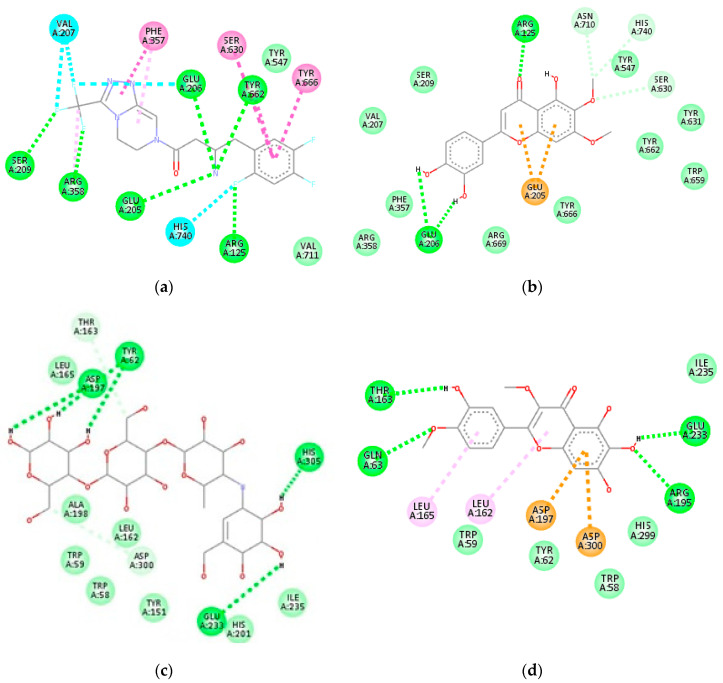
Two-dimensional interaction diagram of compounds with amino acid residues of DPP-4 and α-Amylase enzymes: (**a**) sitagliptin and DPP-4, (**b**) 5,3′,4′-trihydroxy-6,7-dimethoxy-flavone and DPP-4, (**c**) acarbose and α-Amylase, (**d**) quercetagetin-3,4′-dimethyl ether and α-Amylase.

In this study, molecular docking was used to estimate the binding mechanism of putative compounds in CF4. These were achieved by predicting their respective conformation and binding affinities with DPP-4 and α-Amylase enzymes. Molecular docking is a reliable aid for active compounds’ identification, and a positive correlation has been found between in silico analysis and in vitro results [11]. Prior to docking, redocking was done to validate the docking protocol in this study. The re-docked structures produced a closely similar conformation with that of the X-ray structure. The two-dimensional (2D) interaction diagrams of complex (**8**)-DPP-4 and complex (**7**)-α-Amylase with the lowest binding affinity have been presented in Figure 3. Meanwhile, the two-dimensional interaction diagrams between the other compounds and DPP-4 and α-Amylase can be found in supplementary material Figures S1 and S2, respectively. The binding affinity and interacting residues between the identified compounds and the enzymes are presented in Table 4.

For DPP-4 inhibitory docking analysis, amino acid residues—Ser630, Asn710, His740, Val656, Tyr547, Tyr631, Tyr662, Tyr666, Trp659, Val711, Tyr662, Glu205, Glu206, Arg358, Ser209, and Phe357—were considered critical for the inhibition of the enzyme. The positive control, sitagliptin, bound to the active site of DPP-4 with a binding affinity of −8.6 kcal/mol and showed hydrogen bonding interactions with Ser209, Arg358, Glu205, Arg125, Tyr662, and Glu206. Other significant interactions involving Phe357, Ser630, Tyr666, His740, and Val207 can also be observed (Figure 3a).

All the compounds identified showed interactions with the essential residues of DPP-4, thus suggesting that they contribute to the DPP-4 inhibitory activity of CF4. Among the compounds, compound (**8**) showed the best binding affinity (−8.1 kcal/mol), which is comparable to that of sitagliptin. The 3′-4′ catechol group of ring B of the compound was able to form hydrogen bonding with Glu205 of DPP-4 while the substitutes on the ring A and ring C moieties formed other hydrogen bonds with Arg125, Asn710, His740, and Ser630. The π system of ring A and ring C further formed hydrophobic interaction with Glu205 (Figure 3b). Similar interactions can be observed for other flavonoids where the catechol group of ring B, the π system of ring A and ring C, as well as hydroxyl (-OH) and methoxy (-OCH_3_) substituents played a substantial role in binding interactions with DPP-4. The binding affinity of compound (**5**) (−7.7 kcal/mol) was slightly higher when compared to other flavonoids, which may be due to its higher number of methoxy substituents on ring A and ring B that led to bulkier structure and caused binding hindrance into the cavity.

Compound (**4**) interacted with DPP-4 with a binding affinity of −7.8 kcal/mol. The interactions between (**4**) and the amino acid residues Glu206, Glu205, Asn710, Arg125, and Ser630 of DPP-4 were predominantly credited to the hydroxyl groups of its glucose unit. The glucose moiety in compounds has been shown to assist in DPP-4 inhibitory activity in an earlier study [12]. Another compound that showed comparable binding affinity to that of sitagliptin is compound (**1**) (−7.7 kcal/mol). The important interactions between compound (**1**) and DPP-4 include interactions with Glu206, Arg125, Ser630, Glu205, and Tyr666. Compound (**2**), with the least favorable binding affinity (−5.4 kcal/mol), showed the least interactions with critical residues of DPP-4.

For α-Amylase inhibitory docking analysis, positive control acarbose bound to the active site of α-Amylase with the binding affinity of −6.9 kcal/mol and formed extensive hydrogen-bonding interactions with His305, Glu233, Tyr62, Asp197, Asp300, and Thr163 (Figure 3c). Acarbose is a saccharide that works as an α-Amylase inhibitor drug. Most of the tentatively identified compounds in CF4 showed lower binding affinity values than that of acarbose except for compounds (**2**) (−6.4 kcal/mol) and (**3**) (−6.0 kcal/mol). The higher binding affinity value of the (**3**)-α-Amylase complex could be due to steric hindrance by the ethyl ester chain of the compound. The compound also showed the least interaction with amino acid residues of α-Amylase.

Compound (**7**) showed the lowest binding affinity (−8.6 kcal/mol) with α-Amylase among all the compounds. The -OH and -OCH_3_ substituents of the compound interacts with Thr163, Glu233, Gln63, and Arg195 of α-Amylase via hydrogen bonding, whereas hydrophobic interactions with Leu162, Leu165, Asp197, and Asp300 were due to rings A, B, and C of the compound (Figure 3d). The other flavonoids showed a binding affinity with α-Amylase in the range of −8.0 to −8.4 kcal/mol. Compound (**4**), the structure of which slightly resembles that of acarbose due to its glucose unit, also showed good binding affinity (−8.2 kcal/mol) with α-Amylase. Its glucose moiety formed hydrogen bond interactions with Asp300, His299, Asp197, and Glu233, while the methyl cyclohexene moiety showed hydrophobic interactions with Trp59 and Leu165.

Compound (**1**) also showed a binding affinity of −8.2 kcal/mol. However, more hydrophobic interactions were observed between the compound (**1**) and α-Amylase instead of hydrogen bonding. The compound (**1**) formed hydrophobic interactions with Ala198, Leu162, and His201 and two hydrogen bonding with Glu233 and Asp300. Compound (**9**) showed a higher binding affinity value of −7.5 kcal/mol.

Based on the binding affinity and interacting residues from molecular docking analysis, the tentatively identified compounds in CF4 have shown the potential to be DPP-4 and α-Amylase inhibitors. Compounds (**8**) and (**7**), respectively, showed the lowest binding affinity and interactions comparable to sitagliptin and acarbose, which suggested them as effective DPP-4 and α-Amylase inhibitors. Nevertheless, verification of the compounds’ inhibitory bioactivity against these enzymes by in vitro bioassays would be necessary.

## 3. Materials and Methods

### 3.1. Chemicals and Materials

Column chromatography was carried out using Merck Kieselgel PF254 silica gel, Art. No. 1.07734.1000. Thin-layer chromatography (TLC) was done using Merck DC-Plasticfolie TLC plastic sheet pre-coated with Kieselgel 60 PF (Darmstadt, Germany). Dimethylsulfoxide, hexane, chloroform, acetone, methanol, water, acetonitrile, formic acid, acarbose were purchased from Sigma Aldrich (Saint Louis, MO, USA). All solvents used for LC-MS/MS analysis were HPLC grade; others were analytical grade. The DPP-4 inhibitory screening kit was purchased from Cayman Chemical (Ann Arbor, MI, USA). α-Amylase was purchased from Megazyme (County Wicklow, Ireland).

### 3.2. Plant Materials

The leaves and stem bark of *M. glabra* were collected from Pasir Putih, Kelantan, Malaysia in 2018. The plant material was identified by Dr. Mohd. Firdaus Ismail (Biodiversity Unit, Institute of Bioscience, Universiti Putra Malaysia). A voucher specimen was deposited at the mini herbarium of the Institute of Bioscience, Universiti Putra Malaysia with an accession number of SK3326/18.

### 3.3. Preparation of Extracts and Fractionation

Shade-dried leaves (1.0 kg) and stem bark (1.0 kg) of *M. glabra* were separately ground to a fine powder. The plant materials were macerated with hexane (5 L) at room temperature for 72 h, followed by filtration of the extracts using Whatman No.1 filter paper. The plant residues were re-macerated twice using a fresh batch of hexane solvent and filtered in the same manner. The filtrates collected were pooled and evaporated to dryness using a rotary evaporator at 40 °C (Buchi, Switzerland) to yield crude hexane extracts of leaves and stem bark, respectively. The extraction procedures were successively repeated with chloroform and methanol solvents to yield different crude extracts. All extracts from the leaves and stem bark of *M. glabra* were subjected to antidiabetic assays, namely DPP-4 and α-Amylase inhibitory assays. The implementation of bioassay-guided fractionation of plant extracts was proven to lead to the isolation of bioactive compounds [25]. Bioassay-guided fractionation of chloroform leaves’ crude extract (20.0 g) was performed using glass column chromatography (8 cm × 25 cm) packed with silica gel 60 (70–230 mesh) and eluted with increasing polarities of solvent systems starting from hexane, hexane: acetone, acetone: methanol to methanol. A total of 109 fractions (F1–F109) of 200 mL each were collected from the column chromatography. All the fractions were subjected to the thin layer chromatography (TLC) technique and the fractions with similar TLC profiles were pooled to give five major fractions, CF1 (F1–F23), CF2 (F24–F39), CF3 (F40–F56), CF4 (F57–F73) and CF5 (F74–F109). These major fractions were then subjected to antidiabetic assays.

### 3.4. Determination of Extraction Yield

The extraction yield (%) of *M. glabra* was calculated using the Equation (1):

(1) $$Extraction\;yield\;\left(\%\right)\;=\;\left[\frac{dry\;weight\;of\;extract}{dry\;weight\;of\;sample}\right]\;\times \;100$$

### 3.5. Antidiabetic Assays

#### 3.5.1. DPP-4 Inhibitory Assay

DPP-4 inhibitory activity of test samples was investigated using a DPP-4 inhibitor screening kit. Initially, 30 μL of diluted assay buffer and 10 μL of diluted human-recombinant DPP-4 enzyme solution were pipetted and mixed into each well of a 96-well plate containing 10 μL of samples with different concentrations in dimethylsulfoxide. Next, 50 μL of the diluted fluorogenic substrate, Gly-Pro-Aminomethylcoumarine (AMC), was added to initiate the reaction. The 96-well plate was incubated at 37 °C for 30 min. After incubation, the excitation and emission fluorescence of free AMC was measured at 350–360 nm and 450–465 nm, respectively, by using a microplate reader (BioTek Instruments, Inc., Winooski, VT, USA). For the negative and positive control wells, dimethylsulfoxide solvent and sitagliptin standard were used, respectively. The percentage of inhibition was calculated using the Equation (2):

(2) $$\%\;Inhibition\;=\;\left[\frac{OD\;initial\;activity-OD\;inhibitor}{OD\;initial\;activity}\right]\;\times \;100$$

#### 3.5.2. α-Amylase Inhibitory Assay

The α-Amylase inhibition assay was conducted according to the method described by Abdullah et al. with slight modifications [26]. Initially, quantities of 40 μL of the test samples with varying concentrations (0.078–5 mg/mL) were mixed with 30 μL of 0.1 M sodium phosphate buffer in a 96-well microplate prior to the addition of 10 μL of α-Amylase (1 U/mL) into the wells. The plate was subjected to incubation at 37 °C for 15 min, followed by addition of 30 μL of soluble starch (1.0%) and re-incubated at 37 °C for 30 min. The reaction in the mixture was halted by adding 30 μL of hydrochloric acid (1.0 M) and 30 μL of iodine reagent. The absorbance was measured at a wavelength of 620 nm. Acarbose and phosphate buffer were used as the positive and negative controls, respectively. The α-Amylase inhibition activity was calculated using the Equation (3) as follows:

(3) $$\%\;Inhibition=\;\left[\frac{OD\;extract-OD\;control}{OD\;extract}\right]\;\times \;100$$

### 3.6. LC-MS/MS Analysis

The most active fraction, CF4, was subjected to phytochemical profiling using LC-MS/MS in order to identify phytochemical components with DPP-4 and α-Amylase inhibitory activities. LC was performed on the ACQUITY UPLC I-Class system from Waters (Milford, MA, USA), consisting of a binary pump, a vacuum degasser, an auto-sampler, and a column oven. Chromatography separation of phenolic compounds in the fraction was achieved using an ACQUITY UPLC HSS T3 column (100 mm × 2.1 mm × 1.8 μm), also from Waters, kept constant at 40 °C. Two components (A and B) were used for linear binary gradient solvent systems. Component A was water with 0.1% formic acid while component B was acetonitrile. The mobile phase composition was changed during the run in the order of: 0 min, 1% B; 0.5 min, 1% B; 16.00 min, 35% B; 18.00 min, 100% B; 20.00 min, 1% B. The flow rate was maintained at 0.6 mL/min with 1 μL of injection volume.

The UPLC system was coupled to a Vion IMS QTOF hybrid mass spectrometer from Waters, equipped with a Lock Spray ion source for MS/MS characterization. The ion source was set to operate in the mode of negative electrospray ionization (ESI) under the following specific parameters: reference capillary voltage, 3.00 kV; capillary voltage, 1.50 kV; source temperature, 120 °C; desolvation gas flow, 800 L/h; desolvation gas temperature, 550 °C; and cone gas flow, 50 L/h. For desolvation and cone gas, Nitrogen (>99.5%) was employed. Data were acquired in high-definition MSE (HDMSE) mode in the range *m*/*z* 50–1500 at 0.1 s/scan. Hence, two independent scans with different collision energies were alternatively acquired during the run: a low-energy scan at a fixed collision energy of 4 eV, and a high-energy scan where the collision energy was ramped from 10 to 40 eV. Argon (99.999%) was used as collision-induced-dissociation gas. UNIFI Software (Waters) was used to process and analyze the MS data. The comparisons of mass spectra, retention time, parent ion, and MS/MS fragment ions with compounds in the library were performed for the compound identification.

### 3.7. In Silico Inhibitory Analysis of Identified Compounds

**Figure 4:**
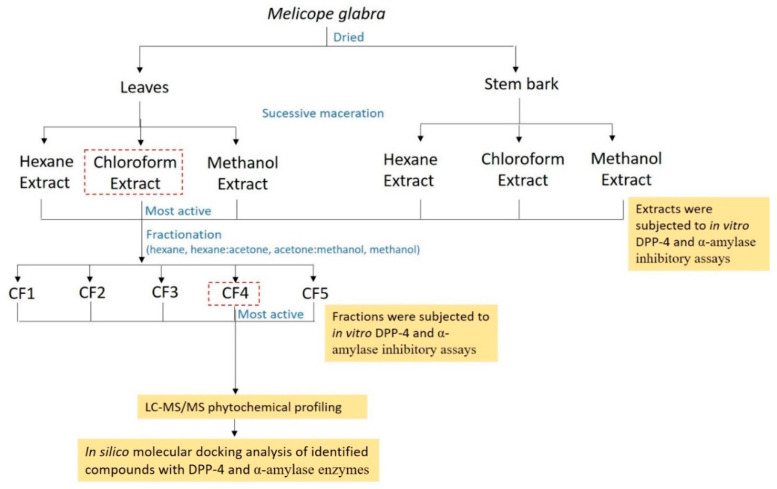
Schematic diagram of the overall flow of the experiment conducted on *M. glabra*.

The x-ray crystal structure of human DPP-4 complexed with the drug sitagliptin (PDB ID 1 × 70) and human pancreatic α-Amylase complexed with acarbose derived pentasaccharide (PDB ID 3BAJ) were retrieved from the Protein Data Bank (https://www.rcsb.org/pdb). The coordinate files were subjected to AutodockTools (ADT) ver. 1.5.6 for the preparation of molecular docking. For each protein structure, the unwanted chains, water molecules, and non-polar hydrogens were removed and polar hydrogens were added. ChemDraw 16.0 software (PerkinElmer Informatics, Waltham, MA, USA) was used to recoup the molecular structure of ligands. ADT was used to assign Gasteiger charges for all atoms involved in the docking. Molecular docking for all ligands was carried out using the Autodock Vina with the grid map size of 60 × 60 × 60 and a grid point spacing of 0.375 Å [27]. The docking results were analyzed using Discovery Studio Visualizer software. The graphical methodology shown in Figure 4 represents an overall flow of experiments conducted in this study.

### 3.8. Statistical Analysis

All experiments were carried out in triplicate. The results are expressed as mean ± standard deviation and differences between means have been statistically analyzed using the *t*-test to compare two treatments. *p* < 0.05 is considered significant. Statistical analysis was performed with GraphPad Prism (San Diego, CA, USA).

## 4. Conclusions

The antidiabetic properties of various extracts from different plant parts of *M. glabra* were described and compared for the first time. The results indicated that *M. glabra* contains diverse polarity bioactive compounds that work on the different mechanisms against diabetes mellitus. Phytochemical profiling on the most active fraction CF4 of chloroform leaves’ extract using LC-MS/MS tentatively identified nine phenolic compounds that may be responsible for the antidiabetic properties of *M. glabra* based on in silico docking analysis. The respective binding affinity and interactions of 5,3′,4′-trihydroxy-6,7-dimethoxy-flavone (**8**) and quercetagetin-3,4′-dimethyl ether (**7**) with DPP-4 and α-Amylase were comparable to those of sitagliptin and acarbose, suggesting these compounds to be effective DPP-4 and α-Amylase inhibitors. The findings from this study revealed the potential of *M. glabra* as a natural and alternative source of antidiabetic compounds. Additional studies involving antidiabetic assays on purified compounds of *M. glabra* are suggested to confirm the responsible bioactive compounds.

## Figures and Tables

**Table 1:** The extraction yield of various *M. glabra* extracts.

Plant Part	Extracts	Extraction Yield
		(%)
Leaves	Hexane	2.03 ± 0.31 ^c^
	Chloroform	2.79 ± 0.19 ^d^
	Methanol	7.30 ± 1.10 ^e^
Stem bark	Hexane	0.3 ± 0.16 ^a^
	Chloroform	0.7 ± 0.03 ^b^
	Methanol	2.13 ± 0.25 ^c^

All values are expressed as mean ± standard deviation of triplicates. Data with different superscript (a–e) are considered significant (*p* < 0.05).

**Table 2:** DPP-4 and α-Amylase inhibitory activities of various *M. glabra* extracts and the chloroform leaves’ fractions.

Plant Part	Extracts	IC_50_ (μg/mL)	
		DPP-4	α-Amylase
Leaves	Hexane	1623.60 ± 121.61 ^g^	4230.12 ± 324.76 ^h^
	Chloroform	169.40 ± 9.30 ^c^	303.64 ± 10.10 ^c^
	Methanol	1086.48 ± 142.69 ^f^	2488.13 ± 231.54 ^g^
Stem bark	Hexane	8408.36 ± 102.23 ^i^	5447.01 ± 243.16 ^i^
	Chloroform	332.31 ± 10.07 ^d^	975.80 ± 17.10 ^e^
	Methanol	4992.33 ± 0.84 ^h^	3946.12 ± 143.21 ^h^
Fractions			
CF1		1711.06 ± 70.32 ^g^	8663.12 ± 110.75 ^j^
CF2		619.31 ± 9.21 ^e^	253.30 ± 19.21 ^b^
CF3		313.18 ± 20.92 ^d^	531.44 ± 11.38 ^d^
CF4		128.35 ± 12.77 ^b^	170.19 ± 20.66 ^a^
CF5		711.42 ± 10.26 ^e^	1817.83 ± 209.42 ^f^
Sitagliptin		0.01 ± 0.01 ^a^	-
Acarbose		-	188.60 ± 14.31 ^a^

“-”: Not available. All values are expressed as mean ± standard deviation of triplicates. Data with a different superscript (a–j) in the same column are considered significant (*p* < 0.05).

**Table 3:** The phytoconstituents were putatively identified in fraction CF4 of *M. glabra* chloroform leaves.

Compound	RT (min)	Identification	Molecular Formula	Observed Neutral Mass (Da)	Mass Error (mDa)	Precursor ion [M−H] (*m*/*z*)	Major Fragments (*m*/*z*)
**Flavonoid**							
5	13.82	4′,5,6,7-tetramethoxy-flavone	C_19_H_18_O_6_	342.1103	0.8	341.1032	311.0925
6	14.07	Isorhamnetin	C_16_H_12_O_7_	316.0583	−0.8	315.0503	300.0270, 272.0280
7	14.22	quercetagetin-3,4′-dimethyl ether	C_17_H_14_O_8_	346.0689	0.4	345.0617	330.0380, 315.0416, 164.9826
8	14.91	5,3′,4′-trihydroxy-6,7-dimethoxy-flavone	C_17_H_14_O_7_	330.0740	0.4	329.0662	314.0427, 299.0192, 271.0244
**Cinnamic acid derivative**							
3	11.91	methyl 3,4,5-trimethoxycinnamate	C_13_H_16_O_5_	252.0998	−0.4	251.0923	193.0506, 179.0352
**Glucoside**							
4	13.04	Renifolin	C_18_H_24_O_7_	352.1522	0.4	351.1452	199.0990
**Lactone**							
2	11.63	swermirin	C_10_H_10_O_4_	194.0579	−0.4	193.0506	179.0352, 149.0611
9	15.15	2-methoxy-5-acetoxy-fruranogermacr-1(10)-en-6-one	C_18_H_24_O_5_	320.1624	0.7	319.1554	305.1400, 179.0356
**Coumarin**							
1	10.20	*trans*-decursidinol	C_14_H_14_O_5_	262.0841	−0.2	261.0765	203.0349, 159.0460

**Table 4:** Binding affinity and interacting residues of the identified compounds with DPP-4 and α-Amylase enzyme.

Compounds	DPP-4			α-Amylase		
	Binding Affinity (kcal/mol)	H-Bond	Hydrophobic	Binding Affinity (kcal/mol)	H-Bond	Hydrophobic
1	−7.7	Glu206, Arg125, Ser630, Glu205	Glu205, Tyr666	−8.2	Glu233, Asp300	Ala198, Leu162, His201
2	−5.4	Lys122, Asp739	His740, Arg125	−6.4	His299, Asp197, Tyr62, Asp300	Ala198, Trp58
3	−5.6	Arg125, Tyr547, Arg669, Val 207, Tyr662	Tyr666, Glu205	−6.0	His299, Tyr62, Thr163	Trp59
4	−7.8	Glu206, Glu205, Asn710, Arg125, Ser630	Tyr547, Phe357	−8.2	Asp300, His299, Asp197, Glu233, His305	Trp59, Leu165
5	−7.7	Arg125, Ser630, His740, Asn710, Tyr547, Val207	Glu205, Phe357	−8.4	His299, Gln63, Asp300, Glu233	Tyr62, Trp59
6	−7.8	Glu206, Arg358, Tyr547	Phe357, Tyr666	−8.0	Lys200, Tyr151, Asp197	Ile235, Ala198, His201, Leu162, Lys200
7	−7.9	Ser209, Arg125, Tyr631	Glu205, Glu206, Tyr666, Phe662	−8.6	Thr163, Glu233, Gln63, Arg195	Leu162, Leu165, Asp197, Asp300
8	−8.1	Glu206, Arg125, Asn710, His740, Ser630	Glu205	−8.1	Glu233, Asp197, Asp300	Trp59, Leu165
9	−6.3	His126, Arg125	Glu205, Tyr666, Phe357	−7.5	Gln63, Asp197	Leu162, His299, Tyr62, His305, Trp59, Trp58
sitagliptin	−8.6	Ser209, Arg125, Arg358, Glu205, Glu206, Tyr 662	Phe357, Ser630, Tyr666, Val207, His740	-	-	-
acarbose	-	-	-	−6.9	His305, Glu233, Tyr62, Asp197, Asp300, Thr163	-

## Supplementary Materials

The following are available online, Figure S1: 2D interaction diagram of identified compounds with amino acid residues of DPP-4—(**a**) 4′,5,6,7-tetramethoxy-flavone, (**b**) quercetagetin-3,4′-dimethyl ether, (**c**) isorhamnetin, (**d**) methyl 3,4,5-trimethoxycinnamate, (**e**) renifolin, (**f**) 2-methoxy-5-acetoxy-fruranogermacr-1(10)-en-6-one, (**g**) swermirin, (**h**) *trans*-decursidinol; Figure S2: 2D interaction diagram of identified compounds with amino acid residues of α-Amylase—(**a**) 4′,5,6,7-tetramethoxy-flavone, (**b**) 5,3′,4′-trihydroxy-6,7-dimethoxy-flavone, (**c**) isorhamnetin, (**d**) methyl 3,4,5-trimethoxycinnamate, (**e**) renifolin, (**f**) 2-methoxy-5-acetoxy-fruranogermacr-1(10)-en-6-one, (**g**) swermirin, (**h**) *trans*-decursidinol.

## Sample Availability

Samples of the leaves and stem bark crude extracts are available from the authors.
