# Supplementary material for: Identification of Dipeptidyl Peptidase-4 and α-Amylase Inhibitors from Melicope glabra (Blume) T. G. Hartley (Rutaceae) Using Liquid Chromatography Tandem Mass Spectrometry, In Vitro and In Silico Methods
Source: Molecules. 2020 Dec 22;26(1):1. doi: 10.3390/molecules26010001 (PMC7792625; doi:10.3390/molecules26010001)
Supplement: Supplementary file 1 [file molecules-26-00001-s001.pdf]

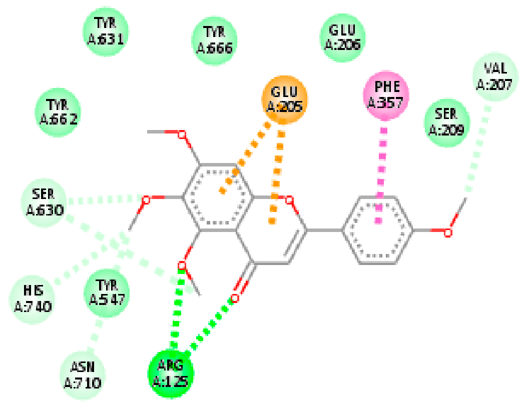

TYR A:631
TYR A:666
GLU A:206
VAL A:207
TYR A:662
GLU A:205
PHE A:357
SER A:209
SER A:630
HIS A:740
TYR A:547
ASN A:710
ARG A:125

(a)

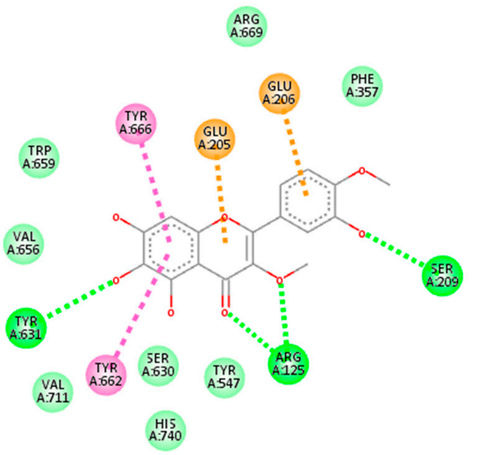

ARG A:669
GLU A:206
PHE A:357
TYR A:666
GLU A:205
TRP A:659
VAL A:656
SER A:209
TYR A:631
TYR A:662
SER A:630
TYR A:547
ARG A:125
VAL A:711
HIS A:740

(b)

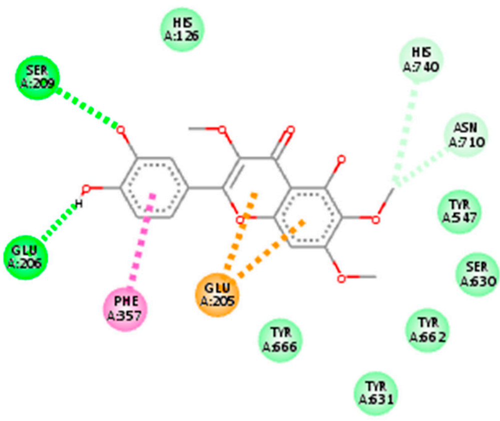

HIS A:126
SER A:209
HIS A:740
ASN A:710
TYR A:547
GLU A:206
SER A:630
PHE A:357
GLU A:205
TYR A:666
TYR A:662
TYR A:631

(c)

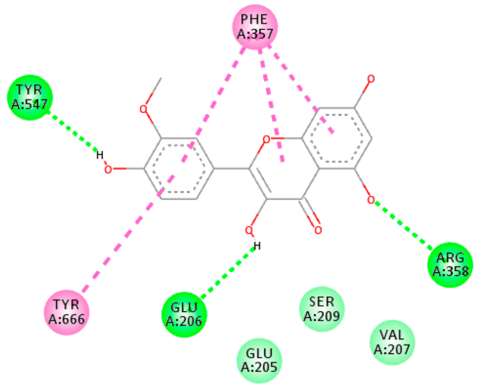

PHE A:357
TYR A:547
ARG A:358
TYR A:666
GLU A:206
SER A:209
VAL A:207
GLU A:205

(d)

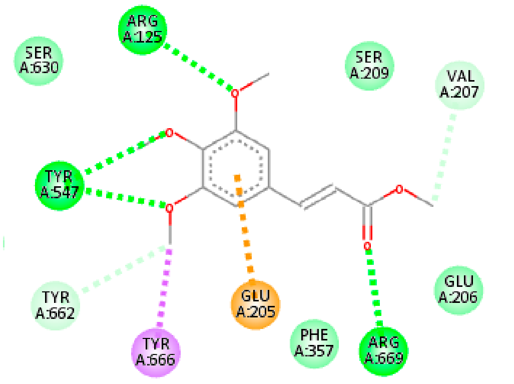

SER A:630
ARG A:125
SER A:209
VAL A:207
TYR A:547
TYR A:662
GLU A:205
GLU A:206
TYR A:666
PHE A:357
ARG A:669

(e)

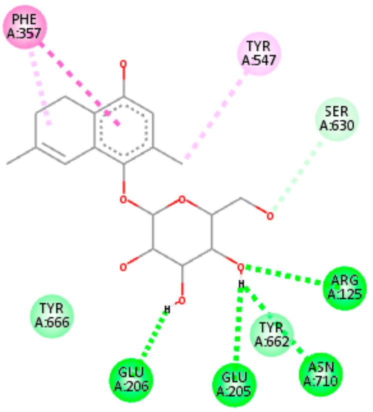

PHE A:357
TYR A:547
SER A:630
ARG A:125
TYR A:666
TYR A:662
GLU A:206
GLU A:205
ASN A:710

(f)

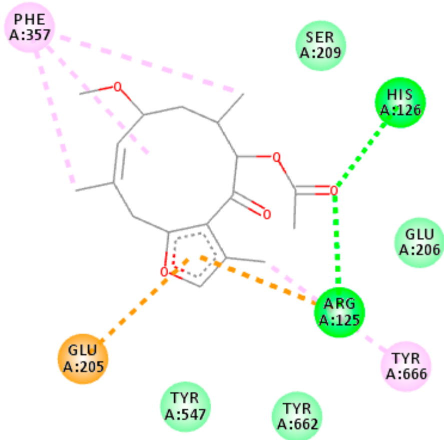

(g)

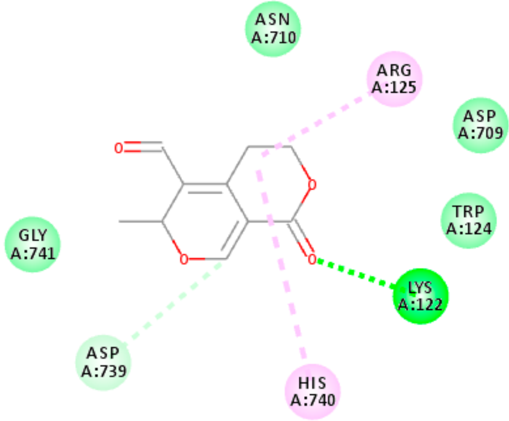

(h)

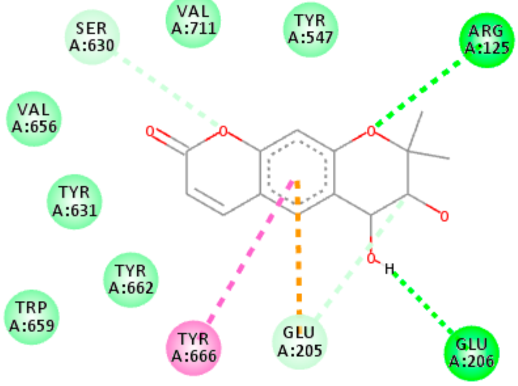

(i)

Figure S1. Two dimensional (2D) interaction diagram of identified compounds with amino acid residues of DPP-4: (**a**) 4',5,6,7-tetramethoxy-flavone (**b**) quercetagetin-3,4'-dimethyl ether (**c**) 3,6,7-trimethylquerce-tagetin (**d**) isorhamnetin (**e**) methyl 3,4,5-trimethoxycinnamate (**f**) renifolin (**g**) 2-methoxy-5-acetoxy-fruranogermacr-1(10)-en-6-one (**h**) swermirin (**i**) *trans*-decursidinol

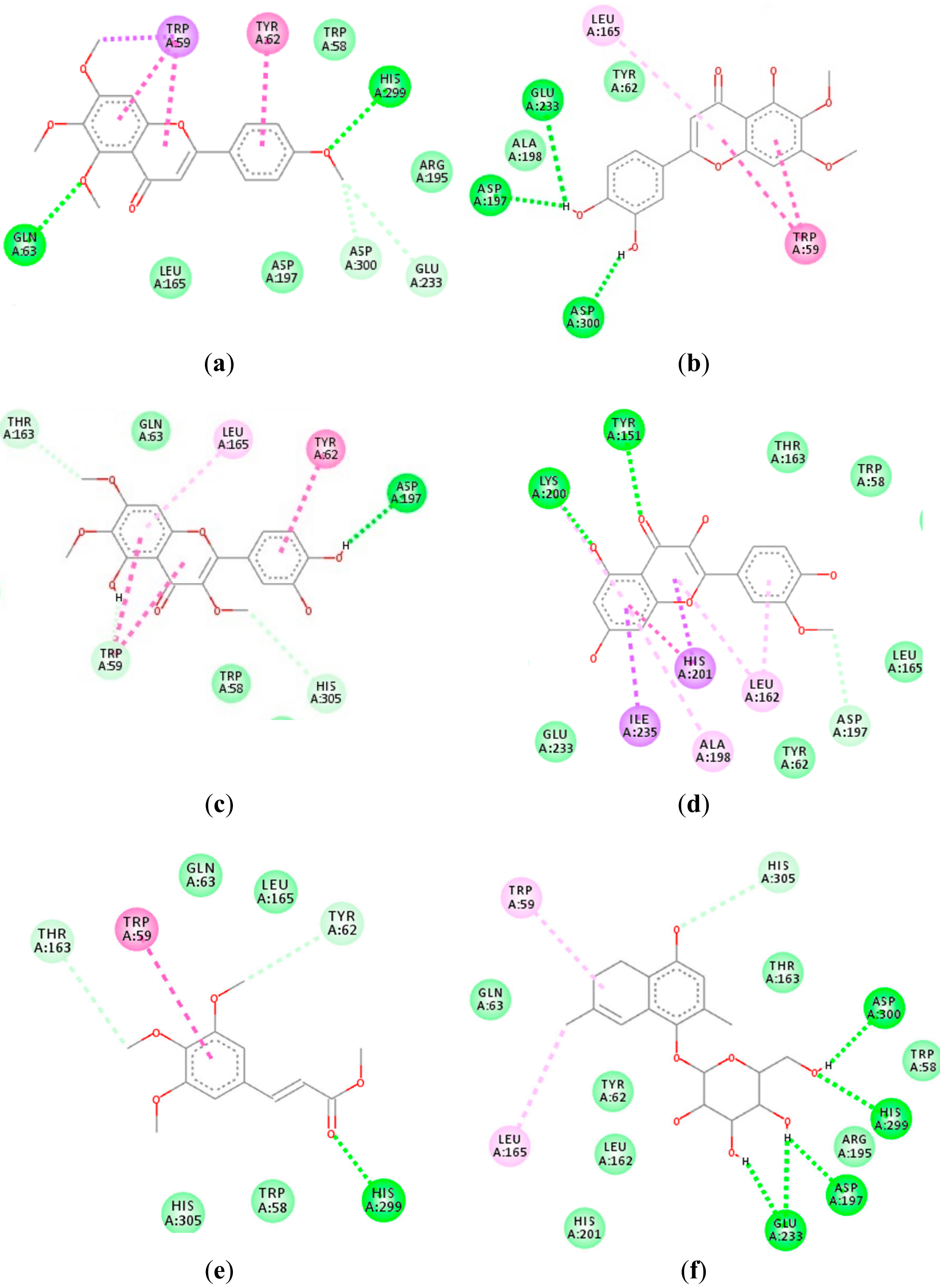

TRP A:59
TYR A:62
TRP A:58
HIS A:299
ARG A:195
GLN A:63
LEU A:165
ASP A:197
ASP A:300
GLU A:233
(a)
LEU A:165
TYR A:62
GLU A:233
ALA A:198
ASP A:197
TRP A:59
ASP A:300
(b)
THR A:163
GLN A:63
LEU A:165
TYR A:62
ASP A:197
TRP A:59
TRP A:58
HIS A:305
(c)
TYR A:151
LYS A:200
THR A:163
TRP A:58
LEU A:165
HIS A:201
LEU A:162
ILE A:235
ALA A:198
GLU A:233
TYR A:62
ASP A:197
(d)
GLN A:63
LEU A:165
TYR A:62
THR A:163
TRP A:59
HIS A:305
TRP A:58
HIS A:299
(e)
HIS A:305
TRP A:59
THR A:163
GLN A:63
ASP A:300
TRP A:58
TYR A:62
HIS A:299
ARG A:195
LEU A:165
LEU A:162
ASP A:197
HIS A:201
GLU A:233
(f)

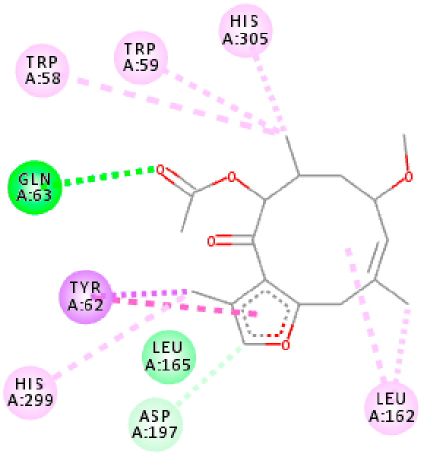

(**g**)

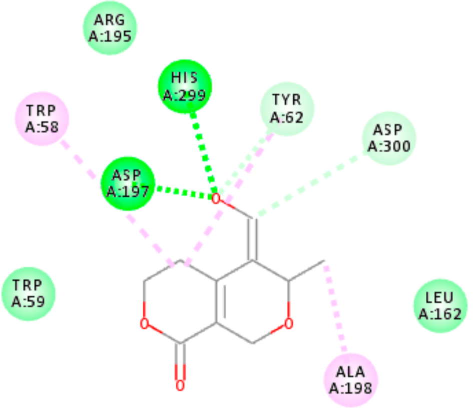

(**h**)

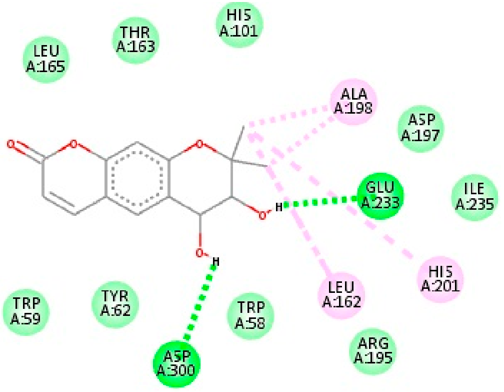

(**i**)

Figure S2. Two dimensional (2D) interaction diagram of identified compounds with amino acid residues of α-amylase: (**a**) 4',5,6,7-tetramethoxy-flavone (**b**) 5,3′,4′-trihydroxy-6,7-dimethoxy-flavone (**c**) 3,6,7-trimethylquerce-tagetin (**d**) isorhamnetin (**e**) methyl 3,4,5-trimethoxycinnamate (**f**) renifolin (**g**) 2-methoxy-5-acetoxy-fruranogermacr-1(10)-en-6-one (**h**) swermirin (**i**) *trans*-decursidinol
